# Anaplastic Thyroid Carcinoma: Current Treatments and Potential New Therapeutic Options with Emphasis on TfR1/CD71

**DOI:** 10.1155/2014/685396

**Published:** 2014-07-01

**Authors:** Rosalba Parenti, Lucia Salvatorelli, Gaetano Magro

**Affiliations:** ^1^Department of Bio-Medical Sciences, Physiology Section, University of Catania, Viale A. Doria 6, 95125 Catania, Italy; ^2^Department G.F. Ingrassia, Section of Anatomic Pathology, University of Catania, Via S. Sofia 87, 95123 Catania, Italy

## Abstract

Anaplastic thyroid carcinoma (ATC) is one of the most aggressive human cancers. Actually, ATC is refractory to conventional therapies, including surgery, chemotherapy, radiotherapy, and radioiodine (^131^I) therapy. Accordingly, genetic and molecular characterizations of ATC have been frequently and periodically reviewed in order to identify potential biological markers exploitable for target therapy. This review briefly focuses on main molecular events that characterize ATC and provides an update about preclinical studies. In addition, the overexpression of transferrin receptor 1 (TfR1/CD71) by neoplastic cells of ATC is emphasized in that it could represent a potential therapeutic target. In this regard, new therapeutic approaches based on the use of monoclonal or recombinant antibodies, or transferrin-gallium-TfR1/CD71 molecular complexes, or lastly small interfering RNAs (siRNAs) are proposed.

## 1. Introduction

Thyroid cancer represents the most frequent malignancy among all endocrine tumors [1]. Well-differentiated thyroid carcinomas, including papillary (PTC) and follicular (FTC) carcinomas, are characterized by a favorable prognosis, while undifferentiated/anaplastic carcinoma (ATC) is an uncommon and highly aggressive form, which usually results in the death of the patient [2–4]. The 5-year survival ranges from 0 to 14%, with a median survival of 2–6 months [5–9]. ATC arises more commonly in female patients, with a mean age of 70 years, usually affected by nodular goiters or with a history of well-differentiated thyroid carcinoma or with nodal or distant metastases [3]. The patients usually complain of hoarseness due to a large-sized and rapidly expanding neck mass, which, at the time of presentation, is often surgically unresectable due to the invasion of surrounding thyroid structures, such as the laryngeal nerve, esophagus and trachea, and/or documentation of distant metastases [3]. The most important prognostic factor is the degree of the extent of disease at diagnosis. Small-sized ATCs or foci of ATC arising in the context of well-differentiated thyroid carcinomas have a better prognosis [9–11]. Obviously the prognosis also depends on the ability to eradicate the disease by surgery [7, 12]. In fact, if the eradication surgery is associated with radiotherapy and adjuvant or neoadjuvant chemotherapy with doxorubicin, survival may slightly increase [7, 9, 13–15]. Unfortunately wide surgical resection usually fails to provide benefits due to the local spread of tumor, while tracheostomy is often performed to ensure the patent of upper airway, invaded and/or obstructed by massive tumor [3]. Grossly, thyroid parenchyma is widely or completely replaced by a fleshy mass, whitish in color, with multiple areas of necrosis and hemorrhage, which diffusely infiltrates adjacent tissues [3, 5, 6]. Histologically, the tumor is composed of a variable mixture of spindled, epithelioid, and large pleomorphic/bizarre giant cells exhibiting different growth patterns such as solid, trabecular, and fascicular patterns [2, 3, 5, 6, 10]. The overall appearance of ATC is usually closely reminiscent of a high-grade pleomorphic sarcoma. Mitotic figures are frequently observed, including atypical mitoses. Hemorrhage and necrosis, sometimes with palisading configuration, are often seen [10]. There may be an inflammatory infiltrate, predominantly of granulocytes, which occasionally can invade the cytoplasm of tumor cells. Although the above mentioned features represent the common basic morphological aspects of ATC, several morphological variants have been described over time, some of which appear to be rather uncommon [16]: (i) squamous cell carcinoma variant (tumor consisting of dominant/pure squamous differentiation); (ii) adenosquamous carcinoma variant (in addition to squamous differentiation, tumor contains foci of glandular differentiation with mucin production); (iii) lymphoepithelioma-like carcinoma variant (tumor sharing morphological features with the nasopharyngeal undifferentiated carcinoma); (iv) rhabdoid variant (tumor exhibits cells with clear-cut rhabdoid morphology); (v) osteoclastic variant (tumor contains reactive CD68+ osteoclast-like multinucleated giant cells intermixed to cancer cells); (vi) carcinosarcoma variant (tumor with a mixture of carcinomatous and heterologous mesenchymal components, such as cartilage, bone, or skeletal differentiation); (vii) paucicellular variant (hypocellular tumor with diffuse sclerosis, mimicking Riedel thyroiditis); (viii) angiomatoid variant (tumor mimicking angiosarcoma). Despite the poor morphological differentiation, the epithelial nature of ATC is demonstrable in 45–80% of cases by staining for cytokeratins, especially using cytokeratin AE1/AE3. Approximately half of the cases express epithelial membrane antigen (EMA). Only rarely there is TTF-1 expression, while thyroglobulin is almost invariably negative. Notably, a significant expression of TP53 is commonly observed [16].

As ATC is refractory to conventional chemotherapy, radiotherapy, and radioiodine (^131^I) therapy [17], new therapeutic approaches are urgently needed in the future. In this regard, some original or review articles about genetic mutations, chromosomal instability, and identification of potential biomarkers exploitable against ATC are emerging in the literature [17–24]. However, while for PTC several potential gene and protein therapeutic targets have been identified [25–29], only a few options seem to be available for ATC in the literature [30]. Waiting for the advent of new genomewide approaches, such as next-generation sequencing (NGS), the analysis of the molecular mechanisms involved in the pathogenesis of ATC still remains the only available tool for planning any target therapy. There is increasing evidence that follicular cell-derived thyroid carcinomas represent a biological continuum of the same disease that progresses from the curable well-differentiated thyroid carcinomas (PTC and FTC) to fatal ATC. In fact, although ATC may derive* de novo*, many cases seem to arise from preexisting PTC or FTC [31–33]. This is supported by morphological evidence showing the gradual loss of papillary and follicular growth patterns associated with a concurrent increase in the presence of solid growth pattern, mitoses, necrosis, and nuclear pleomorphism that is typically observed in ATC. Moreover, most of ATCs exhibit residual foci of differentiated thyroid carcinoma, including both PTC and FTC [16]. Notably, ATC may also develop as a recurrence months or years after the removal of a well-differentiated neoplasm [5, 34]. Apart from this morphological evidence, it has been previously demonstrated that the development of chromosomal instability underlies the progression to more aggressive phenotypes of thyroid cancer [35]. Recurrent gains at 3p13-14 and 11q13 and loss of 5q11–31 were identified exclusively in ATC, suggesting they may be markers for anaplastic transformation [35].

For ATC with minor PTC or FTC components, it is likely that the mutations typically occurring in the latter tumors (e.g., RAS and BRAF mutations) may represent only early events in the tumorigenesis of ATC, while others, including TP53, catenin beta 1, and PIK3CA, may contribute later to the acquisition of a phenotype responsible for the extremely aggressive behavior of ATC [32, 36–38].

Generally, the genes coding proteins differently involved in the transduction pathway, such as RET, RAS, BRAF, PI3K, PTEN, and AKT, are mutated or aberrantly expressed in ATC, providing conditions for uncontrolled cellular proliferation and carcinogenesis via the MAP kinase pathway. RAS point mutations involving specific regions (codons 12, 13, and 61) of the three RAS oncogenes, H-RAS, K-RAS, and N-RAS, by activating both the MAP kinase pathway and the PI3K/AKT pathway, are associated with aggressive thyroid tumor phenotypes including ATC [39–42].* BRAF*, which belongs to the RAF family of serine/threonine kinases, by regulating the MAP kinase/ERKs signaling pathway, affects cell division, differentiation, and secretion. The most frequent BRAF mutation involves nucleotide 1799 and results in substitution of valine for glutamate at residue 600 (V600E). This point mutation leads to constitutive activation of BRAF kinase and chronic stimulation of the MAPK pathway, playing tumorigenic activity for thyroid cells [30, 32, 43]. Inhibition of BRAF V600E by using vemurafenib has shown promising clinical responses in metastatic PTC [44]. Although BRAF mutation (V600E) is reported in approximately only 25% of ATC, suggesting its involvement in tumor progression together with other genetic markers, it could be exploitable as potential therapeutic target. In this regard a dramatic response to vemurafenib has been obtained in a 51-year-old man with BRAF-mutated anaplastic thyroid cancer [45]. This single case report provides evidence for testing ATCs for BRAF mutation (V600E), treating the positive cases by using vemurafenib. This approach could be suggested, at least, as empirical treatment in rapidly progressive cases. Anyway, the results need to be confirmed in larger series of ACTs.

Different alterations of PTEN/PI3K/AKT pathway that regulates several cellular processes, including cell cycle progression, adhesion, and motility, are also commonly observed in ATC, and then they could be exploitable as potential therapeutic targets. In this regard the missense mutations of* PIK3CA*, which encodes the p110*α* catalytic subunit of phosphatidylinositol 30-kinase (PI3K), have been frequently detected [32, 38, 46]. Aberrant activation of PI3K/Akt pathway has been suggested to promote progression of a thyroid adenoma to FTC and/or ATC [47], while activation of Akt has been observed in most of the ATCs with PIK3CA mutation [32, 38, 46].

Molecular mechanisms involved in tumor cell dedifferentiation are thought to be mediated by loss/inactivation or mutation of tumor suppressor gene,* p53* [30, 32, 36, 38, 48–51]. It has been suggested that, unlike RAS and BRAF gene alterations, p53 mutations are crucial in accelerating genomic instability, triggering tumor dedifferentiation toward ATC [36, 51]. Redifferentiation of tissues from ATC upon the reintroduction of wild type p53 and the restoration of cellular responsiveness to physiologic stimuli, such as thyroid stimulating hormone and reexpression of thyroid peroxidase [38, 52], strongly support this hypothesis.

The biological process of dedifferentiation from well-differentiated thyroid carcinomas toward ATC is also underlined by *β* *-catenin* expression. *β*-Catenin acts as cell-cell adhesion molecule that complexes with E-cadherin proteins in normal epithelium. Derangement of the E-cadherin/catenin complex, as well as low membrane *β*-catenin expression or its nuclear localization, is associated with transformation of differentiated carcinomas into ATC [38, 53–57].

New diagnostic and therapeutic opportunities are emerging by the analysis of* microRNAs* (miRNAs). miRNAs are a heterogeneous class of small noncoding but functional RNAs involved in posttranscriptional regulation of target genes, playing a control role in development, proliferation, apoptosis, and stress response [58, 59]. As miRNA expression is frequently altered in several tumors, they are recently emerging as promising prognostic biomarkers and therapeutic agents for many tumors [60, 61]. Specific miRNA profiles have been associated with thyroid tumors [62–66]. Visone et al. [63] by performing miRNA-chip-microarray analysis demonstrated aberrant miRNA expression profile, especially decrease of some of them (miRNAs-30d, -125b, -26a, and 30a-5p), which clearly differentiates ATC from normal thyroid tissues and PTC. Subsequently, Mitomo et al. [64] confirmed downregulation of some miRNAs, such as -26a and -138, but they also noticed upregulation of others, including miRNAs-21, -146b, -221, and -222. The association of specific miRNAs deregulation with ATC transformation is the rational approach for further challenging investigations. In fact, miRNA-125b has a different expression in the human tumors, being upregulated in pancreas and stomach carcinomas, whereas it is downregulated in breast cancer and ATC, suggesting that it can act in different ways depending on the cellular context [67]. Moreover, miRNA-125b and others, which significantly decreased in ATC [63], have, among the predicted regulated target genes, also HMGA1 and HMGA2, which are proteins expressed at very high levels in several malignant tumors, including thyroid carcinomas [68, 69]. Again, miRNA-21, described to be upregulated [64], targets E2F (involved in cell cycle and apoptosis) and inhibits PTEN; miRNA-138, found to be downregulated [63, 64], targets the human telomerase reverse transcriptase (hTERT) gene which is also found to be totally downregulated in both ATC and PTC cell lines in comparison with normal thyroid tissues [64]. Nevertheless, miRNAs are emerging as promising new strategy with therapeutic potential for many aggressive cancers, such as ATC.

Preclinical studies, through* in vitro* and* in vivo* analyses, are providing helpful information in the therapeutic approach of ATC, especially the analysis of the mouse model closely recapitulating the clinic-pathological features of human ATC. While most genetically engineered mouse models gave significant advancements about differentiated thyroid carcinomas, such as PTC [70–72] and FTC [73–76], only recently ATC mouse models have been developed.

Antico Arciuch et al. [77] firstly obtained a mouse model of ATC by combining, in the mouse thyroid follicular cells, two molecular hallmarks of human ATC, namely, activation of PI3K (via Pten deletion) and inactivation of p53. By the age of 9 months, over 75% of the compound mutant mice developed aggressive, undifferentiated thyroid tumors, displaying all the features of their human counterpart, including pleomorphism, epithelial-mesenchymal transition, aneuploidy, local invasion, and distant metastases. It was shown that the tumors developing in this animal model undergo the glycolytic shift known as Warburg effect and are highly sensitive to the therapeutic use of glycolytic inhibitors, which synergize with standard chemotherapy [77]. Later, Nehs et al. [78] elegantly demonstrated the remarkable efficacy of PLX4720 compound, which is an ATP analog that selectively inhibits B-Raf^V600E^ by stabilizing it in an inactive conformation [79], to induce significant regression in an orthotopic mouse model of ATC even when administered at a very late therapeutic intervention stage. This result seems to be particularly promising. It is well known, in fact, that ATC tends to be resistant to traditional approaches such as standard chemotherapy, radiation, and radioiodine (^131^I) due to loss of the sodium iodide symporter through malignant dedifferentiation [80]. If downregulation of BRaf with anti-BRaf^V600E^ therapy also causes sodium iodide symporter upregulation, as suggested by* in vitro* data, it could be expected that patients treated with anti-B-Raf^V600E^ therapy may undergo both reduction of tumor size/invasiveness and possible redifferentiation, thus making radioactive iodine administration possible to control an additional metastatic burden [78, 81–83]. A detailed description of an approach establishing an orthotopic mouse model of ATC has been reported by Sewell et al. [84], which mainly emphasized thyroid tumor metastasis and disease related cachexia and respiratory distress. Just recently, McFadden et al. [85] have genetically engineered a mouse model of BRAF-mutant ATC and demonstrated that combination treatment with MEK and BRAF inhibitors results in enhanced antitumor activity as compared to treatment with a BRAF inhibitor alone, suggesting that this combination could be useful as a component of treatment regimens also in human.

Given these results, it must be stressed that the animal model is a tool of unquestionable benefit for the development of appropriate therapeutic approach against complex diseases. Orthotopic mouse models seem to be ideal and commonly used for preclinical and translational studies of compounds and therapies, not only because of the fact that they may mimic key aspects of human diseases (e.g., metastasis), but also because of their reproducibility and the possibility to evaluate systemic effects of treatments [86]. Thus, even if aggressive thyroid tumors, such as ATC or poorly differentiated thyroid carcinoma, carry several complex genetic alterations, likely explaining disease progression and resistance to single-compound approaches, orthotopic models of human thyroid cancer also hold the potential to be good models for testing novel combinatorial therapies [86].

## 2. TfR1/CD71: A Potential Therapeutic Target

Among the biomarkers which have been identified in ATC, by using different approaches, and that can be exploitable as potential therapeutic targets, we focus on type I receptor for transferrin (TfR1/CD71) [82]. TfR1, also known as CD71, is a type II cell membrane-associated glycoprotein involved in iron homeostasis and cell growth [87–89]. Although ubiquitously expressed on the cell surface, TfR1/CD71 is commonly upregulated in cells with high proliferative index, including cancer cells that need iron as cofactor of many enzymatic reactions, such as DNA synthesis [87–90]. TfR1/CD71 overexpression has been reported in several human malignant tumors, including lymphomas, carcinomas, neuroendocrine, and brain tumors [91]. Among carcinomas, TfR1/CD71 overexpression has been documented in colon, stomach, pancreas, breast, lung, liver, bladder, oral cavity, and uterus [91]. Notably a close correlation of TfR1/CD71 expression level and tumor proliferation index, histological grading, stage, and prognosis has also been largely demonstrated [87–91].

**Figure 1:**
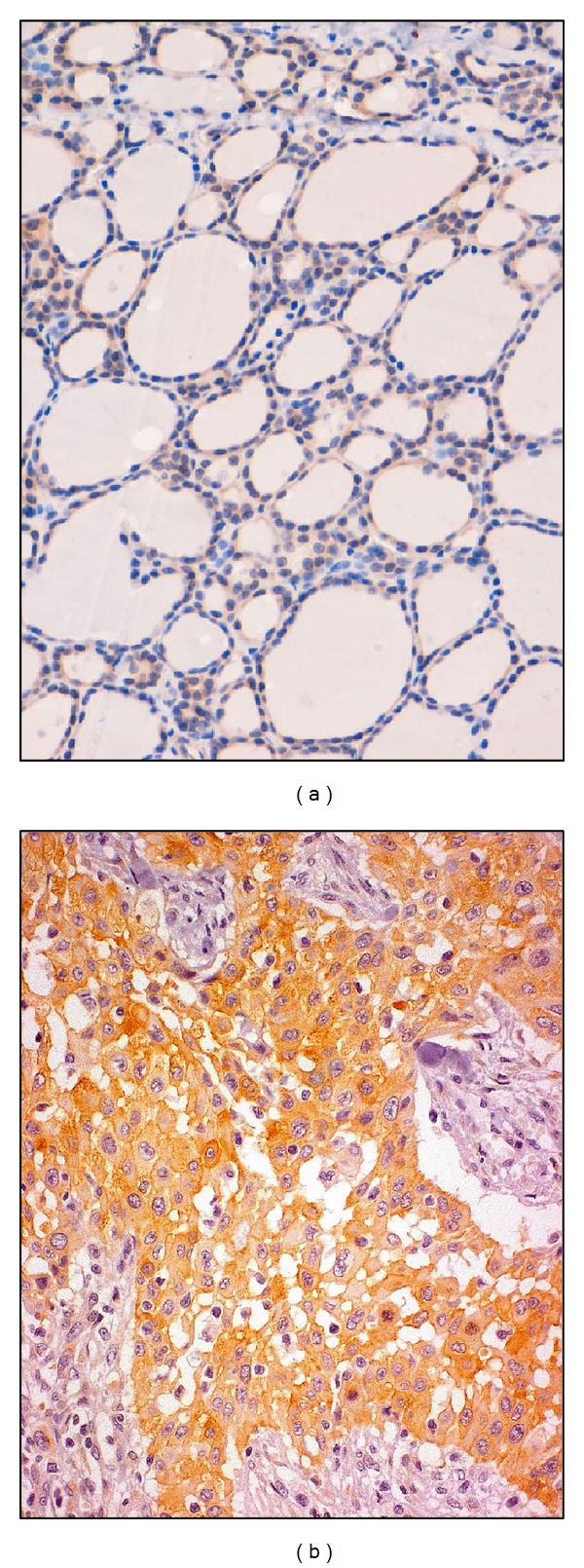
(a) Cells of a nodular goiter showing a weak and cytoplasmic staining for TfR1/CD71. (b) In ATC TfR1/CD71 is diffusely expressed both in the cytoplasm and in the cell surface of neoplastic cells.

**Figure 2:**
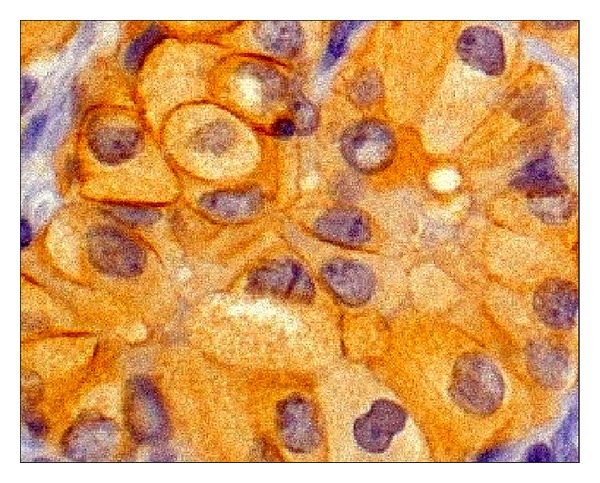
Higher magnification showing concurrent cytoplasmic and cell membrane immunostaining for TfR1/CD71 in ATC.

**Figure 3:**
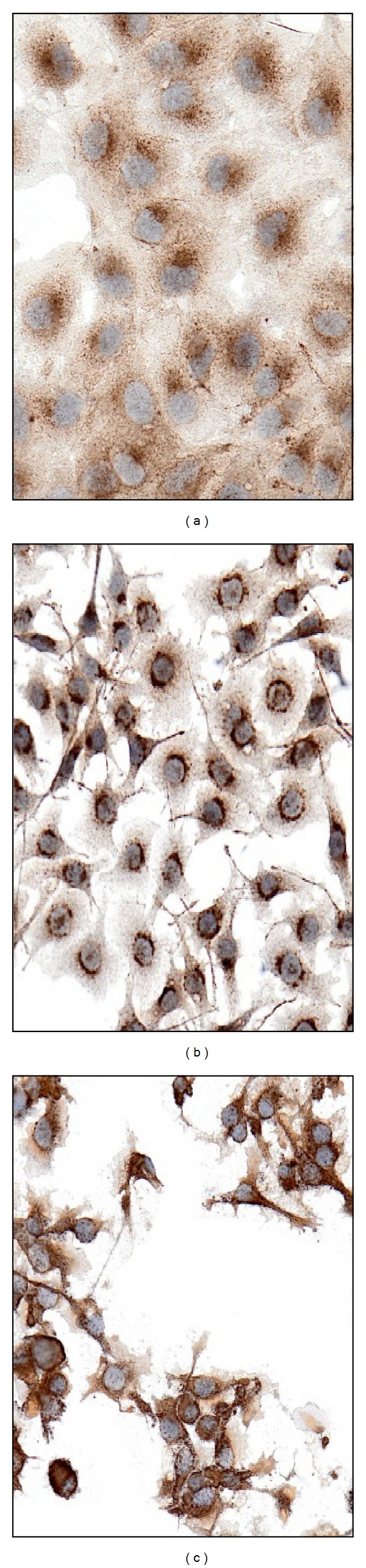
TfR1/CD71 is differentially expressed in thyroid tumor cell lines: increasing cytoplasmic expression is seen in neoplastic cells from PTC (a), FTC (b), and ATC (c).

**Figure 4:**
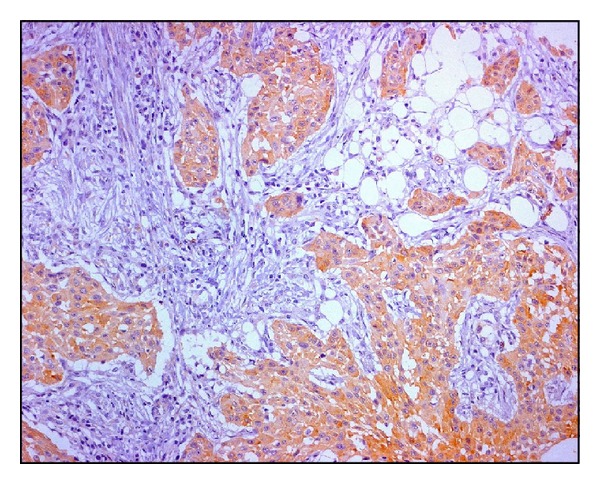
An example of ATC: transferrin is diffusely and strongly expressed in the cytoplasm of neoplastic cells.

Based on our previous studies which showed an increased transferrin expression by PTC cells in comparison with thyroid cells from benign tissues [25], we performed PCR, western blotting, and immunohistochemical studies on fresh, paraffin-embedded thyroid tissues, as well as in thyroid cell lines, to assess whether TfR1/CD71, the receptor for transferrin, is upregulated in malignant thyroid tumors [91]. Conventional RT-PCR revealed the presence of TfR1/CD71 mRNA in all thyroid samples examined, suggesting that this receptor is transcribed in both benign and malignant tissues but differently expressed in malignant versus benign tissues. In fact, western blot analyses showed that although TfR1/CD71 protein was detected in all examined samples, its relative abundance appeared substantially higher in malignant tissues, especially PTC and ATC, when compared to their benign counterparts. Immunohistochemical results paralleled the findings of western blot, revealing an overall overexpression of the receptor in malignant tissues as compared to benign tissues which, by contrast, did not or only weakly and focally showed low levels of expression [91]. All the above mentioned findings suggest that synthesis and membrane incorporation of TfR1/CD71 occur at low levels in normal thyroid tissues, whereas it becomes part of an aberrant gene/protein expression pattern upon neoplastic transformation and malignant progression [91]. In particular, TfR1/CD71 overexpression was observed in all cases of ATCs tested (10 out 10 cases), and similarly to most cases of PTC, a combined strong and diffuse cytoplasmic, as well as, cell membrane immunostaining was observed (Figures 1 and 2) [91]. Similar results were also obtained in ATC cell lines (Figure 3). Our unpublished immunohistochemical data, showing that most neoplastic cells of ATC exhibit strong and diffuse cytoplasmic staining for transferrin (Figure 4), suggest that the overexpression of this protein concurs with the increased expression of its cognate receptor. Thus, it could be speculated that, as already seen in PTC [25], an autocrine and/or paracrine regulatory loop of transferrin-TfR1/CD71 does exist also in ATC. However we admit that this hypothesis needs to be confirmed by mRNA analyses to exclude the possibility that immunohistochemically detected transferrin can derive from internalized protein present in blood or outside the cell through interaction with its receptor. Interestingly, more than 30 years ago, it was largely known that gallium-67 citrate scintigraphy was helpful in identifying malignant thyroid tumors, especially primary and metastatic ATCs [92, 93]. At that time, it was hypothesized that there was a close correlation between gallium-67 uptake and degree of malignancy of thyroid tumor cells, even if the mechanism of tumor localization of gallium-67 was still unclear [92, 93]. Nowadays it is commonly accepted that gallium-67 citrate is preferentially uptaken by high-grade malignant tumors, through its ability to bind, in place of transferrin, TfR1/CD71 [94–96]. This is supported by the evidence that only malignant tumors that overexpressed TfR1/CD71 at a tissue level were also marked* in vivo* by gallium [97–99]. Based on our findings, it is likely that the high uptake of gallium-67 citrate by ATC cells might be explained by the high expression of TfR1/CD71 in this aggressive neoplasm.

**Figure 5:**
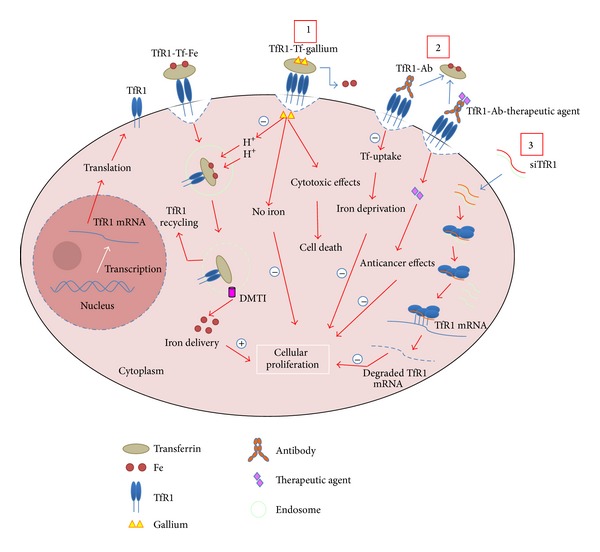
The diagram shows cellular uptake of iron by internalizing the transferrin-iron complex through TfR1-mediated endocytosis and summarizes the main therapeutic strategies through TfR1. 1: gallium binds avidly to transferrin, competing with iron (Fe), to form transferrin-gallium complexes, which in turn binds TfR1/CD71 on the surface of neoplastic cells containing high density of TfR1. Gallium, delivered in the cytoplasm, may be capable of interfering with the intracellular release of iron from the endosome to cellular compartments. Gallium also has direct cytotoxic effects resulting in cell death. 2: monoclonal antibody (Ab) against TfR1 binds TfR1 on the cell surface overexpressing the receptor, like in tumor cells, by competing or not with Fe-transferrin complexes depending on the location of the epitope on the receptor to which the antibody binds. This activity, in turn, results in iron deprivation and cell proliferation inhibition. Other types of antibodies have been engineered to deliver therapeutic agents with anticancer effects. 3: small interfering RNA (siRNA) approach. siRNA, alone or complexed, by transit across cellular membrane, are delivered to cytoplasm, where by classical Dicer-RISC-pathway-unwinding-mRNA recognition-cleavage-mRNA degradation could drive TfR1 downregulation. Both three proposed mechanisms potentially conduct to the reduction of iron in the cytoplasm of neoplastic cell and thus to the inhibition of cellular proliferation.

Because of its upregulation in malignant tissues, extracellular accessibility, and constitutive ability to internalize into cells, TfR1/CD71 is currently attracting wide interest as potential direct or indirect therapeutic target [87–89, 91]. Firstly, TfR1/CD71 can be targeted by direct interaction with conjugates of its ligand transferrin (Tf), the iron transporting protein. In this regard, gallium nitrate binds avidly to transferrin to form transferrin-gallium complexes, which in turn bind TfR1/CD71 on the surface of neoplastic cells [94, 100] (Figure 5). Gallium nitrate or alternatively gallium compounds, a group IIIa metal salt, have been described to inhibit the proliferation of tumor cells* in vitro* and* in vivo* [101]. Antitumoural activity by novel organogallium (III) through induction of apoptosis has also been described in 8505C anaplastic thyroid cancer cell line [102].

Gallium nitrate cytotoxicity may be due to its capability to interfere with the release of iron from endocytic vesicles, depending on not only TfR1/CD71 receptor density on cellular surface but also TfR1/CD71 cycling [95]. In addition, cytotoxicity of gallium may be due, at least in part, to the inhibition of iron uptake. Gallium nitrate binds transferrin and thus, iron cannot bind and is not taken up by the cell [103, 104]. Thus, the transferrin-gallium-TfR1/CD71 molecular complex may represent a promising therapeutic approach against ATC.

TfR1/CD71 can be also specifically targeted by monoclonal or recombinant antibodies (Figure 5). In this regard, there are two different types of antibodies: (i) directly cytotoxic antibodies and (ii) therapeutic agents delivery antibodies. The former, binding TfR1, inhibit the function of the receptor by inducing its sequestration and subsequently degradation in sensitive cells. It has been shown that TfR1 level reduction on the cell surface results in decreased transferrin uptake and induction of lethal iron deprivation (LID) in hematopoietic malignancies [105, 106].

Other monoclonal or recombinant antibodies have been developed to target TfR1/CD71 for delivering chemotherapeutic drugs, protein toxins, radionuclides, liposomes, modified viral particles, and nanoparticles to kill malignant cells [87–89, 107, 108] (Figure 5). The combinations of such antibodies against TfR on human tumor cells have been demonstrated to have antiproliferative effects both* in vitro* and* in vivo* [106, 109–114].

Lastly, TfR1/CD71 may be exploitable as specific target for small interfering RNA (siRNA) approach (Figure 5). This technology represents a powerful genetic tool for sequence-specific inhibition of target proteins capable of modulating cell growth, apoptosis, chemoresistance, and chemosensitivity. In this regard the use of transferrin should ensure specific targeting of siRNA-containing complexes to ATC cells in situ and the consequent uptake by TfR1/CD7-mediated endocytosis of the delivered particles. Recently a siRNA clinical trial has successfully targeted nanoparticles containing transferrin, which engage TfR on the surface of cutaneous melanoma cells [115]. However, the targeting specificity reported in this study has been questioned by other authors who failed to demonstrate the overexpression of TfR1/CD71 in a large series of cutaneous melanomas [116]. Accordingly it was contemplated the possibility that neoplastic cells might internalize nanoparticles conjugated with transferrin through a mechanism independent of the activity of the cognate receptor [116].

In conclusion, although these potential TfR1/CD71-based therapeutic strategies appear to be intriguing, the question of whether this receptor will remain accessible* in vivo* in ATC is still to be elucidated. For these reasons we advise that future* in vitro* and preclinical studies will be performed to confirm the idea of using TfR1/CD71 as a meaningful molecule for target therapy against ATC, which continues to be one the most aggressive human tumors.

## References

[1] Enewold L, Zhu K, Ron E (2009). Rising thyroid cancer incidence in the United States by demographic and tumor characteristics, 1980–2005. *Cancer Epidemiology Biomarkers and Prevention*.

[2] Patel KN, Shaha AR (2006). Poorly differentiated and anaplastic thyroid cancer. *Cancer Control*.

[3] Cornett WR, Sharma AK, Day TA (2007). Anaplastic thyroid carcinoma: an overview. *Current Oncology Reports*.

[4] Wein RO, Weber RS (2011). Anaplastic thyroid carcinoma: palliation or treatment?. *Current Opinion in Otolaryngology and Head and Neck Surgery*.

[5] Spires JR, Schwartz MR, Miller RH (1988). Anaplastic thyroid carcinoma. Association with differentiated thyroid cancer. *Archives of Otolaryngology—Head and Neck Surgery*.

[6] Lo C, Lam K, Wan K (1999). Anaplastic carcinoma of the thyroid. *The American Journal of Surgery*.

[7] Haigh PI, Ituarte PH, Wu HS (2001). Completely resected anaplastic thyroid carcinoma combined with adjuvant chemotherapy and irradiation is associated with prolonged survival. *Cancer*.

[8] McIver B, Hay ID, Giuffrida DF (2001). Anaplastic thyroid carcinoma: a 50-year experience at a single institution. *Surgery*.

[9] Sugitani I, Kasai N, Fujimoto Y, Yanagisawa A (2001). Prognostic factors and therapeutic strategy for anaplastic carcinoma of the thyroid. *World Journal of Surgery*.

[10] Aldinger KA, Samaan NA, Ibanez M, Stratton Hill C (1978). Anaplastic carcinoma of the thyroid. A review of 84 cases of spindle and giant cell carcinoma of the thyroid. *Cancer*.

[11] Demeter JG, De Jong SA, Lawrence AM, Paloyan E (1991). Anaplastic thyroid carcinoma: risk factors and outcome. *Surgery*.

[12] Venkatesh YS, Ordonez NG, Schultz PN, Hickey RC, Goepfert H, Samaan NA (1990). Anaplastic carcinoma of the thyroid. A clinicopathologic study of 121 cases. *Cancer*.

[13] Nilsson O, Lindeberg J, Zedenius J (1998). Anaplastic giant cell carcinoma of the thyroid gland: treatment and survival over a 25-year period. *World Journal of Surgery*.

[14] Voutilainen PE, Multanen M, Haapiainen RK, Leppäniemi AK, Sivula AH (1999). Anaplastic thyroid carcinoma survival. *World Journal of Surgery*.

[15] Pierie J-PEN, Muzikansky A, Gaz RD, Faquin WC, Ott MJ (2002). The effect of surgery and radiotherapy on outcome of anaplastic thyroid carcinoma. *Annals of Surgical Oncology*.

[16] Ordonez N, Baloch Z, Matias-Guiu X, De Lellis RA, Lloyd RV, Heitz PU, Eng C (2004). Undifferentiated (anaplastic) carcinoma. *Pathology and Genetics—Tumor of Endocrine Organs*.

[17] Regalbuto C, Frasca F, Pellegriti G (2012). Update on thyroid cancer treatment. *Future Oncology*.

[18] Salvatore G, Nappi TC, Salerno P (2007). A cell proliferation and chromosomal instability signature in anaplastic thyroid carcinoma. *Cancer Research*.

[19] Sanders EM, LiVolsi VA, Brierley J, Shin J, Randolph GW (2007). An evidence-based review of poorly differentiated thyroid cancer. *World Journal of Surgery*.

[20] Wiseman SM, Masoudi H, Niblock P (2007). Anaplastic thyroid carcinoma: expression profile of targets for therapy offers new insights for disease treatment. *Annals of Surgical Oncology*.

[21] Denaro N, Nigro CL, Russi EG (2013). The role of chemotherapy and latest emerging target therapies in anaplastic thyroid cancer. *Oncotargets and Therapy*.

[22] Deshpande HA, Roman S, Sosa JA (2013). New targeted therapies and other advances in the management of anaplastic thyroid cancer. *Current Opinion in Oncology*.

[23] Guerra A, Di Crescenzo V, Garzi A (2013). Genetic mutations in the treatment of anaplastic thyroid cancer: a systematic review. *BMC Surgery*.

[24] Li M, Milas M, Nasr C (2013). Anaplastic thyroid cancer in young patients: a contemporary review. *The American Journal of Otolaryngology*.

[25] Magro G, Perissinotto D, Schiappacassi M (2003). Proteomic and postproteomic characterization of keratan sulfate-glycanated isoforms of thyroglobulin and transferrin uniquely elaborated by papillary thyroid carcinomas. *American Journal of Pathology*.

[26] Magro G, Schiappacassi M, Perissinotto D (2003). Differential expression of mucins 1–6 in papillary thyroid carcinoma: evidence for transformation-dependent post-translational modifications of MUC1 *in situ*. *Journal of Pathology*.

[27] Wreesmann VB, Sieczka EM, Socci ND (2004). Genome-wide profiling of papillary thyroid cancer identities MUC1 as an independent prognostic marker. *Cancer Research*.

[28] Delys L, Detours V, Franc B (2007). Gene expression and the biological phenotype of papillary thyroid carcinomas. *Oncogene*.

[29] Rusinek D, Sylwia-Ulczok S, Jarzab B (2011). Gene expression profile of human thyroid cancer in relation to its mutational status. *Journal of Molecular Endocrinology*.

[30] Lee J, Hwang JA, Lee EK (2013). Recent progress of genome study for anaplastic thyroid cancer. *Genomics & Informatics*.

[31] Hunt JL, Tometsko M, LiVolsi VA, Swalsky P, Finkelstein SD, Barnes EL (2003). Molecular evidence of anaplastic transformation in coexisting well-differentiated and anaplastic carcinomas of the thyroid. *The American Journal of Surgical Pathology*.

[32] Smallridge RC, Marlow LA, Copland JA (2009). Anaplastic thyroid cancer: molecular pathogenesis and emerging therapies. *Endocrine-Related Cancer*.

[33] Amico P, Lanzafame S, Li Destri G (2010). Warthin tumor-like papillary thyroid carcinoma with a minor dedifferentiated component: report of a case with clinicopathologic considerations. *Case Reports in Medicine*.

[34] Ozaki O, Ito K, Mimura T, Sugino K, Ito K (1999). Anaplastic transformation of papillary thyroid carcinoma in recurrent disease in regional lymph nodes: a histologic and immunohistochemical study. *Journal of Surgical Oncology*.

[35] Wreesmann VB, Ghossein RA, Patel SG (2002). Genome-wide appraisal of thyroid cancer progression. *The American Journal of Pathology*.

[36] Nikiforov YE (2004). Genetic alterations involved in the transition from well-differentiated to poorly differentiated and anaplastic thyroid carcinomas. *Endocrine Pathology*.

[37] Ruggeri RM, Campenni A, Baldari S, Trimarchi F, Trovato M (2008). What is new on thyroid cancer biomarkers. *Biomarker Insights*.

[38] O'Neill JP, Shaha AR (2013). Anaplastic thyroid cancer. *Oral Oncology*.

[39] Garcia-Rostan G, Zhao H, Camp RL (2003). Ras mutations are associated with aggressive tumor phenotypes and poor prognosis in thyroid cancer. *Journal of Clinical Oncology*.

[40] Nikiforov YE (2008). Thyroid carcinoma: molecular pathways and therapeutic targets. *Modern Pathology*.

[41] Nikiforova MN, E Nikiforov Y (2008). Molecular genetics of thyroid cancer: implications for diagnosis, treatment and prognosis. *Expert Review of Molecular Diagnostics*.

[42] de Biase D, Visani M, Pession A, Tallini G (2014). Molecular diagnosis of carcinomas of the thyroid gland. *Frontiers in Bioscience*.

[43] Gómez Sáez JM (2011). Diagnostic and prognostic markers in differentiated thyroid cancer. *Current Genomics*.

[44] Kim KB, Cabanillas ME, Lazar AJ, Williams MD (2013). Clinical responses to vemurafenib in patients with metastatic papillary thyroid cancer harboring BRAF(V600E) mutation. *Thyroid*.

[45] Rosove MH, Peddi PF, Glaspy JA (2013). BRAF V600E inhibition in anaplastic thyroid cancer. *The New England Journal of Medicine*.

[46] García-Rostán G, Costa AM, Pereira-Castro I (2005). Mutation of the PIK3CA gene in anaplastic thyroid cancer. *Cancer Research*.

[47] Hou P, Liu D, Shan Y (2007). Genetic alterations and their relationship in the phosphatidylinositol 3-kinase/Akt pathway in thyroid cancer. *Clinical Cancer Research*.

[48] Donghi R, Longoni A, Pilotti S, Michieli P, Della Porta G, Pierotti MA (1993). Gene p53 mutations are restricted to poorly differentiated and undifferentiated carcinomas of the thyroid gland. *Journal of Clinical Investigation*.

[49] Fagin JA, Matsuo K, Karmakar A, Tang S-, Koeffler HP (1993). High prevalence of mutations of the p53 gene in poorly differentiated human thyroid carcinomas. *Journal of Clinical Investigation*.

[50] Quiros RM, Ding HG, Gattuso P, Prinz RA, Xu X (2005). Evidence that one subset of anaplastic thyroid carcinomas are derived from papillary carcinomas due to BRAF and p53 mutations. *Cancer*.

[51] Sobrinho-Simões M, Máximo V, Rocha AS (2008). Intragenic mutations in thyroid cancer. *Endocrinology & Metabolism Clinics of North America*.

[52] Moretti F, Nanni S, Farsetti A (2000). Effects of exogenous p53 transduction in thyroid tumor cells with different p53 status. *Journal of Clinical Endocrinology and Metabolism*.

[53] Garcia-Rostan G, Tallini G, Herrero A, D'Aquila TG, Carcangiu ML, Rimm DL (1999). Frequent mutation and nuclear localization of *β*-catenin in anaplastic thyroid carcinoma. *Cancer Research*.

[54] Garcia-Rostan G, Camp RL, Herrero A, Carcangiu ML, Rimm DL, Tallini G (2001). Beta-catenin dysregulation in thyroid neoplasms: down-regulation, aberrant nuclear expression, and CTNNB1 exon 3 mutations are markers for aggressive tumor phenotypes and poor prognosis. *American Journal of Pathology*.

[55] Wiseman SM, Masoudi H, Niblock P (2006). Derangement of the E-cadherin/catenin complex is involved in transformation of differentiated to anaplastic thyroid carcinoma. *American Journal of Surgery*.

[56] Li Y, Zhang X, Polakiewicz RD, Yao T, Comb MJ (2008). HDAC6 is required for epidermal growth factor-induced *β*-catenin nuclear localization. *Journal of Biological Chemistry*.

[57] Rossi ED, Straccia P, Palumbo M (2013). Diagnostic and prognostic role of HBME-1, galectin-3, and *β*-catenin in poorly differentiated and anaplastic thyroid carcinomas. *Applied Immunohistochemistry and Molecular Morphology*.

[58] Bartel DP (2004). MicroRNAs: genomics, biogenesis, mechanism, and function. *Cell*.

[59] He L, Hannon GJ (2004). MicroRNAs: small RNAs with a big role in gene regulation. *Nature Reviews Genetics*.

[60] Hauptman N, Glavac D (2013). MicroRNAs and long non-coding RNAs: prospects in diagnostics and therapy of cancer. *Radiology and Oncology*.

[61] Sethi S, Ali S, Philip PA, Sarkar FH (2013). Clinical advances in molecular biomarkers for cancer diagnosis and therapy. *International Journal of Molecular Sciences*.

[62] Pallante P, Visone R, Ferracin M (2006). MicroRNA deregulation in human thyroid papillary carcinomas. *Endocrine-Related Cancer*.

[63] Visone R, Pallante P, Vecchione A (2007). Specific microRNAs are downregulated in human thyroid anaplastic carcinomas. *Oncogene*.

[64] Mitomo S, Maesawa C, Ogasawara S (2008). Downregulation of miR-138 is associated with overexpression of human telomerase reverse transcriptase protein in human anaplastic thyroid carcinoma cell lines. *Cancer Science*.

[65] Nikiforova MN, Tseng GC, Steward D, Diorio D, Nikiforov YE (2008). MicroRNA expression profiling of thyroid tumors: biological significance and diagnostic utility. *Journal of Clinical Endocrinology and Metabolism*.

[66] Takakura S, Mitsutake N, Nakashima M (2008). Oncogenic role of miR-17-92 cluster in anaplastic thyroid cancer cells. *Cancer Science*.

[67] Volinia S, Calin GA, Liu CG (2006). A microRNA expression signature of human solid tumors defines cancer gene targets. *Proceedings of the National Academy of Sciences of the United States of America*.

[68] Chiappetta G, Bandiera A, Berlingieri MT (1995). The expression of the high mobility group HMGI (Y) proteins correlates with the malignant phenotype of human thyroid neoplasias. *Oncogene*.

[69] Berlingieri MT, Pierantoni GM, Giancotti V, Santoro M, Fusco A (2002). Thyroid cell transformation requires the expression of the HMGA1 proteins. *Oncogene*.

[70] Jhiang SM, Sagartz JE, Tong Q (1996). Targeted expression of the ret/PTC1 oncogene induces papillary thyroid carcinomas. *Endocrinology*.

[71] Knauf JA, Ma X, Smith EP (2005). Targeted expression of BRAFV600E in thyroid cells of transgenic mice results in papillary thyroid cancers that undergo dedifferentiation. *Cancer Research*.

[72] Franco AT, Malaguarnera R, Refetoff S (2011). Thyrotrophin receptor signaling dependence of Braf-induced thyroid tumor initiation in mice. *Proceedings of the National Academy of Sciences of the United States of America*.

[73] Kaneshige M, Kaneshige K, Zhu X (2000). Mice with a targeted mutation in the thyroid hormone *β* receptor gene exhibit impaired growth and resistance to thyroid hormone. *Proceedings of the National Academy of Sciences of the United States of America*.

[74] Suzuki H, Willingham MC, Cheng SY (2002). Mice with a mutation in the thyroid hormone receptor *β* gene spontaneously develop thyroid carcinoma: a mouse model of thyroid carcinogenesis. *Thyroid*.

[75] Miller KA, Yeager N, Baker K, Liao X, Refetoff S, Cristofano ADI (2009). Oncogenic Kras requires simultaneous PI3K signaling to induce ERK activation and transform thyroid epithelial cells in vivo. *Cancer Research*.

[76] Antico-Arciuch VG, Dima M, Liao X-H, Refetoff S, Di Cristofano A (2010). Cross-talk between PI3K and estrogen in the mouse thyroid predisposes to the development of follicular carcinomas with a higher incidence in females. *Oncogene*.

[77] Antico Arciuch VG, Russo MA, Dima M (2011). Thyrocyte-specific inactivation of p53 and Pten results in anaplastic thyroid carcinomas faithfully recapitulating human tumors. *Oncotarget*.

[78] Nehs MA, Nucera C, Nagarkatti SS (2012). Late intervention with anti-BRAF V600E therapy induces tumor regression in an orthotopic mouse model of human anaplastic thyroid cancer. *Endocrinology*.

[79] Tsai J, Lee JT, Wang W (2008). Discovery of a selective inhibitor of oncogenic B-Raf kinase with potent antimelanoma activity. *Proceedings of the National Academy of Sciences of the United States of America*.

[80] Braga-Basaria M, Ringel MD (2003). Clinical review 158: beyond radioiodine: a review of potential new therapeutic approaches for thyroid cancer. *Journal of Clinical Endocrinology and Metabolism*.

[81] Durante C, Puxeddu E, Ferretti E (2007). Brief report: BRAF mutations in papillary thyroid carcinomas inhibit genes involved in iodine metabolism. *Journal of Clinical Endocrinology and Metabolism*.

[82] Riesco-Eizaguirre G, Rodríguez I, de La Vieja A (2009). The BRAFV600E oncogene induces transforming growth factor *β* secretion leading to sodium iodide symporter repression and increased malignancy in thyroid cancer. *Cancer Research*.

[83] Kleiman DA, Buitrago D, Crowley MJ (2013). Thyroid stimulating hormone increases iodine uptake by thyroid cancer cells during BRAF silencing. *Journal of Surgical Research*.

[84] Sewell W, Reeb A, Lin RY (2013). An orthotopic mouse model of anaplastic thyroid carcinoma. *Journal of Visualized Experiments*.

[85] McFadden DG, Vernon A, Santiago PM (2014). p53 constrains progression to anaplastic thyroid carcinoma in a Braf-mutant mouse model of papillary thyroid cancer. *Proceedings of the National Academy of Sciences of the United States of America*.

[86] Antonello ZA, Nucera C (2013). Orthotopic mouse models for the preclinical and translational study of targeted therapies against metastatic human thyroid carcinoma with BRAFV600E or wild-type BRAF. *Oncogene*.

[87] Daniels TR, Delgado T, Rodriguez JA, Helguera G, Penichet ML (2006). The transferrin receptor part I: biology and targeting with cytotoxic antibodies for the treatment of cancer. *Clinical Immunology*.

[88] Daniels TR, Delgado T, Helguera G, Penichet ML (2006). The transferrin receptor part II: targeted delivery of therapeutic agents into cancer cells. *Clinical Immunology*.

[89] Daniels TR, Bernabeu E, Rodríguez JA (2012). The transferrin receptor and the targeted delivery of therapeutic agents against cancer. *Biochimica et Biophysica Acta*.

[90] Pantopoulos K (2004). Iron metabolism and the IRE/IRP regulatory system: An update. *Annals of the New York Academy of Sciences*.

[91] Magro G, Cataldo I, Amico P (2011). Aberrant expression of TfR1/CD71 in thyroid carcinomas identifies a novel potential diagnostic marker and therapeutic target. *Thyroid*.

[92] Higashi T, Watanabe Y, Yamaguchi M (1982). The relationships between the Ga-67 uptake and nuclear DNA Feulgen content in thyroid tumors: concise communication. *Journal of Nuclear Medicine*.

[93] Senga O, Miyakawa M, Shirota H (1982). Comparison of Tl-201 chloride and Ga-67 citrate scintigraphy in the diagnosis of thyroid tumor: concise communication. *Journal of Nuclear Medicine*.

[94] Chitambar CR, Zivkovic Z (1987). Uptake of gallium-67 by human leukemic cells: demonstration of transferrin receptor-dependent and transferrin-independent mechanisms. *Cancer Research*.

[95] Chitambar CR, Wereley JP, Matsuyama S (2006). Gallium-induced cell death in lymphoma: role of transferrin receptor cycling, involvement of Bax and the mitochondria, and effects of proteasome inhibition. *Molecular Cancer Therapeutics*.

[96] Chitambar CR, Purpi DP, Woodliff J, Yang M, Wereley JP (2007). Development of gallium compounds for treatment of lymphoma: gallium maltolate, a novel hydroxypyrone gallium compound, induces apoptosis and circumvents lymphoma cell resistance to gallium nitrate. *Journal of Pharmacology and Experimental Therapeutics*.

[97] Kondo K, Noguchi M, Mukai K (1990). Transferrin receptor expression in adenocarcinoma of the lung as a histopathologic indicator of prognosis. *Chest*.

[98] Tsuchiya Y, Nakao A, Komatsu T, Yamamoto M, Shimokata K (1992). Relationship between gallium 67 citrate scanning and transferrin receptor expression in lung diseases. *Chest*.

[99] Högemann-Savellano D, Bost E, Blondet C (2003). The transferrin receptor: a potential molecular imaging marker for human cancer. *Neoplasia*.

[100] Chikh Z, Ha-Duong NT, Miquel G, El Hage Chahine J (2007). Gallium uptake by transferrin and interaction with receptor 1. *Journal of Biological Inorganic Chemistry*.

[101] Chitambar CR (2012). Gallium-containing anticancer compounds. *Future Medicinal Chemistry*.

[102] Gómez-Ruiz S, Gallego B, Kaluderović MR (2009). Novel gallium(III) complexes containing phthaloyl derivatives of neutral aminoacids with apoptotic activity in cancer cells. *Journal of Organometallic Chemistry*.

[103] Chitambar CR, Seligman PA (1986). Effects of different transferrin forms on transferrin receptor expression, iron uptake, and cellular proliferation of human leukemic HL60 cells. Mechanisms responsible for the specific cytotoxicity of transferrin-gallium. *Journal of Clinical Investigation*.

[104] Bernstein LR (1998). Mechanisms of therapeutic activity for gallium. *Pharmacological Reviews*.

[105] Rodríguez JA, Luria-Pérez R, López-Valdés HE (2011). Lethal iron deprivation induced by non-neutralizing antibodies targeting transferrin receptor 1 in malignant B cells. *Leukemia and Lymphoma*.

[106] Daniels TR, Ortiz-Sánchez E, Luria-Pérez R (2011). An antibody-based multifaceted approach targeting the human transferrin receptor for the treatment of B-cell malignancies. *Journal of Immunotherapy*.

[107] Ng PP, Cruz JSD, Sorour DN (2002). An anti-transferrin receptor-avidin fusion protein exhibits both strong proapoptotic activity and the ability to deliver various molecules into cancer cells. *Proceedings of the National Academy of Sciences of the United States of America*.

[108] Jones DT, Trowbridge IS, Harris AL (2006). Effects of transferrin receptor blockade on cancer cell proliferation and hypoxia-inducible factor function and their differential regulation by ascorbate. *Cancer Research*.

[109] Brooks D, Taylor C, Dos Santos B (1995). Phase Ia trial of murine immunoglobulin a antitransferrin receptor antibody 42/6 1. *Clinical Cancer Research*.

[110] Qing Y, Shuo W, Zhihua W (2006). The in vitro antitumor effect and in vivo tumor-specificity distribution of human-mouse chimeric antibody against transferrin receptor. *Cancer Immunology, Immunotherapy*.

[111] Callens C, Moura IC, Lepelletier Y (2008). Recent advances in adult T-cell leukemia therapy: focus on a new anti-transferrin receptor monoclonal antibody. *Leukemia*.

[112] Rodríguez JA, Luria-Pérez R, López-Valdés HE (2011). Lethal iron deprivation induced by non-neutralizing antibodies targeting transferrin receptor 1 in malignant B cells. *Leukemia & Lymphoma*.

[113] Daniels-Wells TR, Helguera G, Rodríguez JA (2013). Insights into the mechanism of cell death induced by saporin delivered into cancer cells by an antibody fusion protein targeting the transferrin receptor 1. *Toxicology In Vitro*.

[114] Leoh LS, Morizono K, Kershaw KM (2014). Gene delivery in malignant B cells using the combination of lentiviruses conjugated to anti-transferrin receptor antibodies and an immunoglobulin promoter. *Journal of Gene Medicine*.

[115] Davis ME, Zuckerman JE, Choi CHJ (2010). Evidence of RNAi in humans from systemically administered siRNA via targeted nanoparticles. *Nature*.

[116] Perris R, Borghese C, Magro G (2011). Pitfalling in nanomedical targeting of melanoma: a “clinical” case of misdelivered RNAi. *Pigment Cell and Melanoma Research*.

